# How to keep alive the “Clean Your Hands” campaign in a hospital setting: six years after

**DOI:** 10.1186/2047-2994-4-S1-P146

**Published:** 2015-06-16

**Authors:** I Neves, D Peres, F Vieira, I Devesa

**Affiliations:** 1 Infection Control and Antimicrobial Resistance Unit, Unidade Local de Saúde de Matosinhos, Matosinhos, Portugal; 2 Infetious Diseases Unit, Unidade Local de Saúde de Matosinhos, Matosinhos, Portugal

## Introduction

The hand hygiene is the most important practice to prevent healthcare associated infection. Portugal joined the WHO “SAVE LIVES: Clean Your Hands” campaign in October 2008, having developed a national strategy supplying training and didactic tools, providing a database and economic incentives to participating hospitals.

## Objectives

Promotion of persistent good practices in hand hygiene.

## Methods

The implementation of the campaign in this acute care 400 bed hospital was coordinated by the Infection Control and Antimicrobial Resistance Unit and focused on the following actions: (1) formal commitment signed by the administration head and the medical/nurse units heads; (2) implementation of multimodal strategy campaign; (3) audits and correction measures to structural conditions of the units (for example: existence of alcohol-based handrub – ABHR at the point of care); (4) infection control link professionals observer training (replicated in each unit) and subsequent observational study before/after the campaign in 2009 and then annually; (5) annual celebration of Hand Hygiene Day (5 of May) with presentation of small films and games, lectures and poster competition. Results are annually revealed, with benchmarking between units, specific interventions in units with lower compliance rates and compliance rates inclusion in its performance indicators.

## Results

Compliance rates of 61,3%, 74.7% and 78.6% before, after campaign in 2009 and 2014, respectively. In the “first moment”: 53,8%, 69.5% and 73,5 respectively. All the professional categories showed improvement. The ABHR consumption increased from 20,2 in 2010 to 31,3 L/1000 patient-days in 2014.

## Conclusion

A national strategy was fundamental to the implementation of the local campaigns. Contributing factors to this success included a national database with real time results (to motivate professionals); performance indicators (to motivate head units) and continuous tools to remember the good practice, keeping alive the campaign.

## Disclosure of interest

None declared.

